# The role of miRNAs carried by extracellular vesicles in type 2 diabetes and its complications

**DOI:** 10.1111/1753-0407.13456

**Published:** 2023-08-16

**Authors:** Yining Chao, Tianwei Gu, Zhou Zhang, Tianyu Wu, Jin Wang, Yan Bi

**Affiliations:** ^1^ Department of Endocrinology, Endocrine and Metabolic Disease Medical Center, Nanjing Drum Tower Hospital, Affiliated Hospital of Medical School Nanjing University Nanjing China; ^2^ Branch of National Clinical Research Centre for Metabolic Diseases Nanjing China

**Keywords:** diabetic complication, extracellular vesicle, miRNA, type 2 diabetes, 糖尿病并发症, 外泌体, miRNA, 2型糖尿病

## Abstract

Diabetes poses severe global public health problems and places heavy burdens on the medical and economic systems of society. Type 2 diabetes (T2D) accounts for 90% of these cases. Diabetes also often accompanies serious complications that threaten multiple organs such as the brain, eyes, kidneys, and the cardiovascular system. MicroRNAs (miRNAs) carried by extracellular vesicles (EV‐miRNAs) are considered to mediate cross‐organ and cross‐cellular communication and have a vital role in the pathophysiology of T2D. They also offer promising sources of diabetes‐related biomarkers and serve as effective therapeutic targets. Here, we briefly reviewed studies of EV‐miRNAs in T2D and related complications. Specially, we innovatively explore the targeting nature of miRNA action due to the target specificity of vesicle binding, aiding mechanism understanding as well as the detection and treatment of diseases.

## Graphical abstract

Highlights

- This review outlines the history of miRNA discovery and its major advances in the field of metabolism.
- We explore the roles of miRNAs in diabetes‐related cognitive impairment.
- We innovatively propose that miRNAs also act with a certain organ‐targeting specificity due to the organ‐targeting nature of extracellular vesicles, which may aid disease detection and treatment and help interpret the mechanisms beneath.
- This review is highly concise and includes the latest research advances, allowing readers to gain a basic framework of the role of EV‐miRNAs in T2D and its complications quickly.

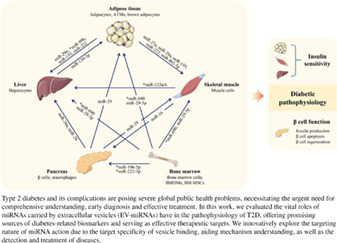

## 摘要

糖尿病是全球严重的公共卫生问题，给社会医疗和经济系统带来沉重负担。其中，90%为2型糖尿病(T2D)。糖尿病经常伴随着严重的并发症，威胁着大脑、眼睛、肾脏和心血管系统等多个器官。由外泌体(EV)携带的miRNA(EV‐miRNA)被认为是介导跨器官和跨细胞通信的关键，并在T2D的病理生理学中发挥重要作用。它们也是糖尿病相关生物标志物的有希望来源，并可作为有效的治疗靶点。在这里，我们简要综述了关于EV‐miRNA在T2D及其相关并发症方面的研究。特别是我们创新性地探讨了miRNA作用的目标特异性，这有助于机制理解和疾病的检测和治疗。

## 1 INTRODUCTION

The worldwide statistics of diabetes are alarming. According to the International Diabetes Federation, 537 million people aged 20–79 years are living with diabetes in 2021, with a projected increase to 783 million by 2045 without effective measures.^1^ Type 2 diabetes (T2D), which is characterized by defective peripheral tissue responsiveness to insulin (insulin resistance, IR) and β cell dysfunction, accounts for ~90% of all diabetes cases. This is highly worrisome given that diabetes often accompanies various complications. It harms the vascular systems while endangering the heart, brain, kidneys, peripheral nerves, eyes, and feet. Diabetes has placed enormous strains on medical and economic systems, necessitating the urgent need for comprehensive understanding, early diagnosis, and effective treatment.

MicroRNAs (MiRNAs) are highly conserved, ubiquitous, small noncoding RNAs (~22 nt in length) generated by practically all cell types. They canonically bind to the 3′ untranslated region (UTR) of mRNAs and perform post‐transcriptional inhibition.^2^ Once considered to act inside the cells, miRNAs are now believed to be encapsulated in extracellular vesicles (EVs) and released into the extracellular environment, hence facilitating intercellular communication and providing indicative information associated with physiological and pathological conditions. Accumulating evidence suggests that EV‐miRNAs are intimately associated with diabetes and its complications. They not only alter under morbid circumstances, offering a promising source of diabetes‐related biomarkers, but also participate in disease development, help us understand the mechanisms beneath, and may serve as effective therapeutic targets.

This review discusses the role of miRNAs carried by EVs in T2D and its complications. First, we outline the discovery history, biosynthesis, and functions of miRNAs, as well as their presence in circulating forms. Then, we illustrate variations of miRNA profiles under diabetic circumstances and the potential prospects they provide as biomarkers. We also demonstrate briefly the respective contributions miRNAs make in the development of diabetes and related complications. After reviewing the latest progress in regulatory mechanisms of EV‐miRNAs, we highlight their prospects as therapeutic avenues. In the last part, current barriers yet to be surmounted for clinical implementation are presented and we pioneeringly explore the potential targeting property of miRNAs due to the targeting nature of vesicles and the contribution this property could make to clinical care.

## 2 miRNAs AND EXTRACELLULAR VESICLES

### 2.1 The history of miRNA discovery

The first hint of miRNA dates back to 1993, when *lin‐4* was discovered to function in the temporal controlling of *C. elegans* development by binding to the 3'UTR of *lin‐14*.^3^, ^4^ In 1997, another gene of the heterochronic pathway, *lin‐28*, was found to bind to and be regulated by *lin‐4*.^5^ This provided a clue regarding the adaptability of this kind of antisense RNA–RNA interaction. It was not until 2000 that another 21‐nucleotide RNA *let‐7* was found.^6^ In the context of the great boom of RNAi research since 1998,^7^ the discovery of *let‐7* identify scientists to take these small RNAs as novel and widespread mRNA regulators.

**FIGURE 1:**
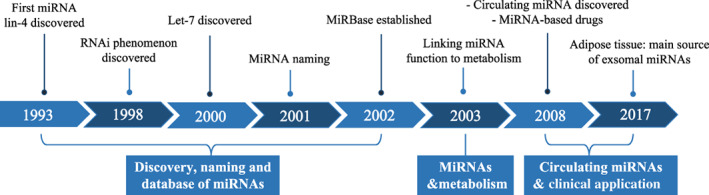
The timeline of miRNA discovery. miRNA, microRNA.

In October 2001, three research teams published their findings in the same issue of *Science*, claiming that *lin‐4* and *let‐7* RNAs belong to a vast class of noncoding RNAs. These were referred to as microRNAs.^8^, ^9^, ^10^ MiRNA research has been in full swing in the years afterward. Hundreds of miRNAs have been found in numerous species including humans, zebrafish, fruit flies and rice.^11^, ^12^ In 2002, miRbase was established to provide a consistent set of guidelines for miRNA identification and naming and a searchable database of published miRNA sequences.^2^, ^13^ Its latest release (v22.1) updated in 2019 includes miRNA sequences from 271 organisms and 48 860 mature miRNAs.^14^ MiRNA research has emerged as one of the most important areas of study in biological science (Figure 1).

### 2.2 Biogenesis and functions of miRNAs

MiRNA biogenesis is a multistep and precise process that undergoes translocation from the cell nucleus to the cytoplasm and requires various enzymes and auxiliary proteins. In metazoans, a canonical process begins with primary miRNA transcripts named pri‐miRNA. With a stem‐loop structure inside, it is cut off in the nucleus by an RNase type III endonuclease Drosha and its cofactor DGCR8. This forms a hairpin structure of about 65 nt, called pre‐miRNA. With help of Exportin 5, pre‐miRNA is exported to the cytoplasm, where it is cleaved again by Dicer. The miRNA/miRNA* duplex is generated and then loaded into the Argonaute protein (AGO), with the less thermodynamically stable strand retained, and the other one degraded. In this way, a single‐strand mature miRNA is finally formed. Afterward, AGO facilitates the assembly of RNA‐induced silencing complex (RISC). RISC mediates the recognition of targeted mRNA, leads to miRNA combination with the 3'UTR of mRNA, and dominates gene silencing by mRNA translational inhibition or degradation.^15^, ^16^, ^17^, ^18^, ^19^

Using loss and gain of function studies, the biological functions of individual miRNAs have been explored widely. It is said that miRNAs involve in the regulation of almost all cellular processes studied to date and are related to physiological and pathological processes like developmental timing, cell differentiation, energy homeostasis, inflammation, tumorigenesis, and organogenesis.^15^, ^20^, ^21^ The first report implicating the involvement of miRNAs in metabolism was done in drosophila in 2003, showing the suppressive effect miR‐14 has on triacylglycerol and diacylglycerol levels.^22^ However, these roles are assumed to be played in their producing cells.^20^

### 2.3 The history of extracellular vesicles

EVs refer to various types of membranous structures secreted by cells and contain bioactive substances including proteins, lipids, and genetic materials such as miRNAs.^23^ EVs are characterized as ectosomes (also known as microparticles or microvesicles), exosomes, and apoptotic bodies based on the size, density, or surface antigen.^24^, ^25^, ^26^, ^27^ However, this classification is imperfect with possible overlap considering the lack of unambiguous physical properties and unified isolating protocols.^28^

Research on EVs can be traced back to the mid‐1940s. Once conceived as “garbage bins” of our bodies, EVs are now believed to be bridges between cell‐to‐cell interactions, participating in cellular signaling and component exchanging. In 2011, the International Society for Extracellular Vesicles was established, and the 2013 Nobel Prize in Physiology or Medicine was awarded to the three scientists who discovered the mechanism regulating vesicular transport.

### 2.4 MiRNAs mediate intracellular crosstalk through EVs

Since 2008, miRNAs have been discovered extracellularly in the circulation and various bodily fluids like saliva, urine, and breast milk.^29^, ^30^ There are two major routines these miRNAs shuttle outside their producing cells. The majority of miRNAs are carried by EVs (EV‐miRNAs), which were elucidated earlier. Although this topic is too extensive to review here, the protein‐miRNA complex is another mode of trafficking in which miRNAs attach to proteins such as high‐density lipoprotein and AGO.^31^ Vesicles and proteins shield extracellular miRNAs from endogenous RNase activity. These miRNAs will then be taken up by recipient cells, unveiling their potential as cell‐to‐cell crosstalk mediators.

EVs can be internalized by recipient cells through membrane fusion, receptor‐dependent endocytosis, micropinocytosis, or phagocytosis.^32^ These mechanisms dictate that the uptake of EVs is relatively targeted and specific. After ingestion by adjacent or distal cells (through circulation), these cargo miRNAs exert paracrine and endocrine functions respectively and mediate a broad range of cellular physiological and pathophysiological processes, including in the process of T2D.

## 3 EV‐miRNAs AND T2D

### 3.1 Distinct circulating miRNA profiles in T2D patients

**FIGURE 2:**
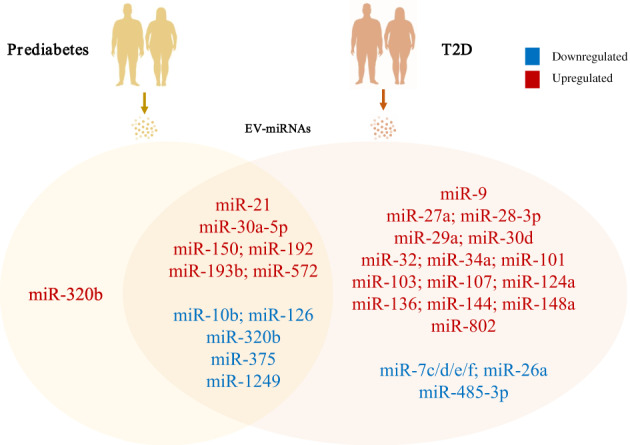
Circulating miRNA alterations under T2D‐related conditions. EV, extracellular vesicles; miRNA, microRNA; T2D, type 2 diabetes.

Several particular miRNAs are reported to express differentially in T2D patients and relate to metabolic parameters. Figure 2 and Table 1 list altered miRNA signatures under different diabetes‐related conditions. Although there is little consensus on miRNA biomarkers, miRNAs like miR‐126 and miR‐34a exhibit consistent trends of variation in different cohorts of T2D patients, providing significant candidates for mechanistic studies and therapeutic options. Meanwhile, levels of miRNAs such as miR‐15a, miR‐146a, miR223, and miR‐375 display inconsistent results, which are probably due to discrepancies in miRNA isolation methods and warrant further investigations. Furthermore, detecting these circulating miRNAs that are differentially expressed in both humans and mice and finding their sources may help guide the establishment of genetic‐manipulating models in mice. To conclude, functional analysis of identified miRNAs provides clues to explore the mechanisms of diabetes and aids translational research in T2D treatment.

**TABLE 1:** Circulating miRNA alterations under T2D‐related conditions.

Year	Condition	MiRNA(s)	Alteration
2010	Diabetic patients, hyperglycemic Lep^ob^ mice^51^	*miR‐15a, miR‐126, miR‐223, *miR‐320	↓
		miR‐28‐3p	↑
2011	Newly diagnosed T2D patients^93^	miR‐9, miR‐29a, miR‐30d, miR‐34a, miR‐124a, *miR‐146a, *miR‐375	↑
2012	T2D patients^94^	miR‐150, miR‐192, miR‐27a, *miR‐320a, *miR‐375	↑
2013	Diabetes patients^49^	miR‐126	↓
2014	Prediabetic and newly diagnosed T2D patients^95^	miR‐126	↓
2015	T2D patients^96^	miR‐103	↑
2015	T2D patients^97^	miR‐101, *miR‐375, miR‐802	↑
2015	Prediabetic patients, glucose‐intolerant mice^98^	miR‐192, miR‐193b	↑
2016	Diabetes patients^52^	miR‐26a, miR‐126	↓
2016	Prediabetes patients^99^	*miR‐146a, miR‐126	↓
	T2D patients^99^	miR‐21, miR‐148a, miR‐30d, miR‐34a	↑
2016	Prediabetic and newly diagnosed T2D patients^100^	miR‐1249, *miR‐320b	↓
		miR‐572	↑
2017	T2D patients^44^	*miR‐15a	↑
2017	Obese T2D patients^101^	let‐7d, let‐7e, let‐7f, let‐7c, miR‐485‐3p	↓
		miR‐144, miR‐193b, miR‐136, miR‐34a, miR‐32	↑
2018	Prediabetic and T2D patients, db/db mice, HFD mice^102^	miR‐27a	↑
2018	Prediabetic and T2D patients^103^	*miR‐15a, *miR‐375	↓
		miR‐150, miR‐30a‐5p	↑
2019	IGT and T2D patients^104^	miR‐21	↑
2020	IGT and T2D patients^57^	*miR‐15a	↑
2020	Prediabetic and T2D patients^105^	miR‐10b	↓
		miR‐223‐3p	↑
2022	T2D patients^106^	miR‐107	↑

Abbreviations: HFD, high‐fat diet; IGT, impaired glucose tolerance; T2D, type 2 diabetes.

* Inconsistent results in different clinical cohorts.

MiRNAs possess characteristics of stability, specificity, and sensitivity and can be retrieved in a relatively noninvasive manner and thus are probably valuable biomarkers. Yet, the separation and identification process must be standardized. A considerable amount of data from clinical samples is required to validate their potential and assist in establishing an appropriate baseline value.

### 3.2 EV‐miRNAs and diabetic pathophysiology

**FIGURE 3:**
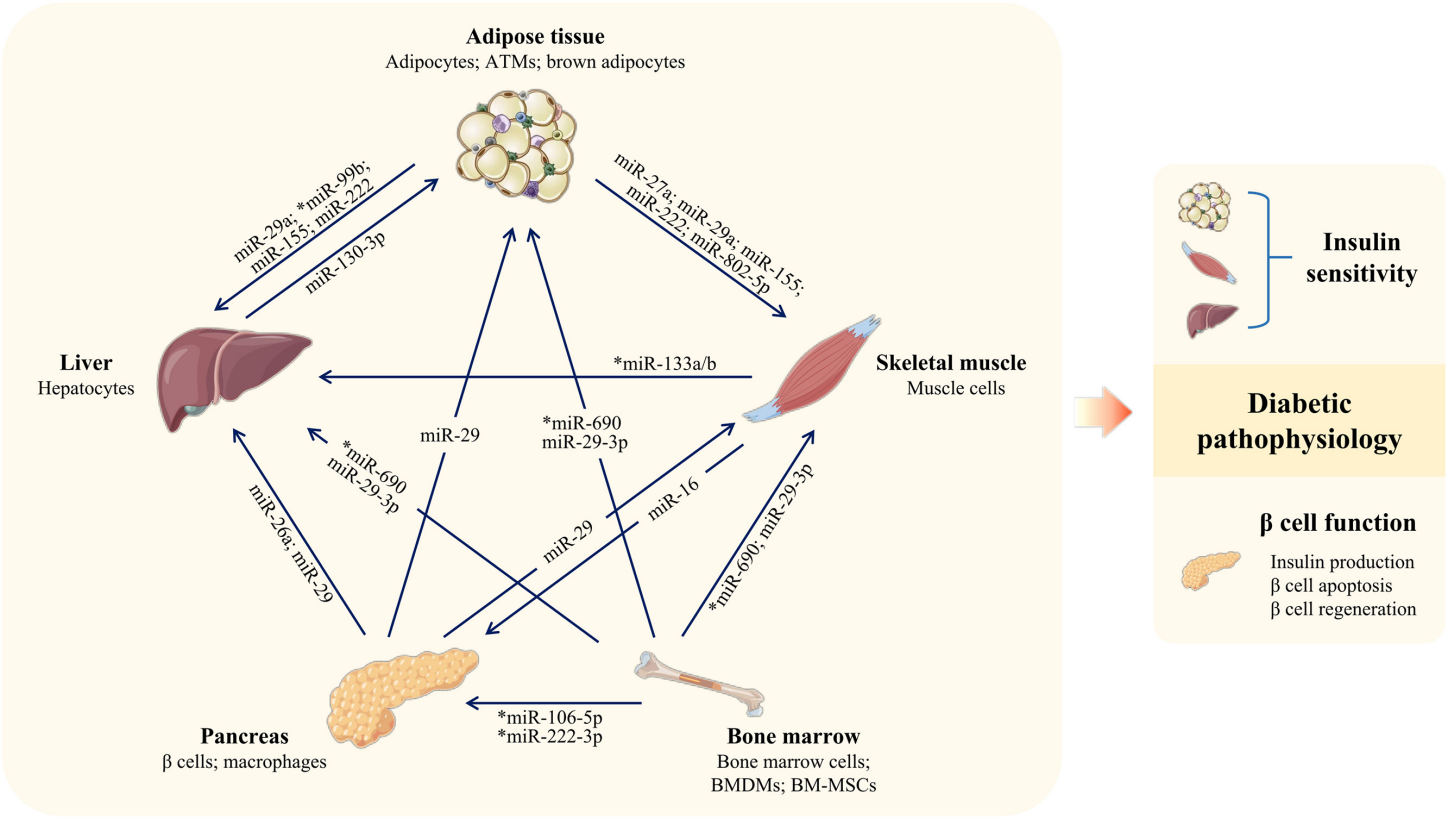
EV‐miRNAs mediate intercellular crosstalk. ATM, adipose tissue macrophage; BMDMs, bone marrow‐derived macrophage; BM‐MSCs, bone marrow mesenchymal stem cells; EV, extracellular vesicles. *miRNAs exerting beneficial effects.

As important intracellular communicating factors, miRNAs from various sources are carried in EVs and trafficked to target cells/organs (Figure 3 and Table 2). By affecting insulin sensitivity and β cell function (including quantity and function), they play proactive or detrimental roles in diabetic progression.

**TABLE 2:** EV‐miRNA‐mediated intercellular crosstalk in T2D pathophysiology.

Source	Forms	miRNA(s)	Expression	Target gene(s)	Recipient cell/organ	Effects
Pancreas						
β cells of obese patients or mice	Exosomes	miR‐26a	↓	Not identified	Hepatocytes	Result in IR and hyperinsulinemia^33^
β cells	Exosomes	miR‐29	↑	TRAF3	Macrophages	Regulates systemic inflammatory response and glucose homeostasis^107^
M1‐like macrophages from islet of HFD mice	Exosomes	miR‐212‐5p	↑	SIRT2	β cells	Impair insulin production^35^
Cytokine‐treated MIN6B1 cells	Exosomes	miR‐7, miR‐29a, miR‐146a	↑	Not identified	Adjacent β cells	Trigger β cell apoptosis^34^
Mouse pancreatic cancer cells	Exosomes	Not identified	↑	Not identified	Skeletal muscle cells	Trigger lipid deposit and impair glucose uptake^108^
Adipose tissue						
BAT	Exosomes	*miR‐99b	↑	FGF21	Liver	Improve glucose tolerance^36^
ATMs of obese mice	Exosomes	miR‐155	↑	PPARγ	Liver, muscle, adipose tissue	Result in glucose intolerance and IR^38^
ATMs of obese mice	Exosomes	miR‐155	↑	SOCS1	Adipocytes	Promote inflammatory response and interfere with insulin signaling^39^
ATMs of obese mice	Exosomes	miR‐29a	↑	PPARδ	Adipocytes, myocytes, hepatocytes	Result in IR^109^
High glucose‐induced ATMs	Exosomes	miR‐210	↑	NDUFA4	3 T3‐L1 adipocytes	Impair glucose uptake in 3 T3‐L1 adipocytes^110^
Adipocytes of HFD mice	Not identified	miR‐27a	↑	PPARγ	C2C12 skeletal muscle cells	Result in IR in muscle^102^
Hypertrophic adipocytes	Exosomes	miR‐802‐5p	↑	HSP60	Cardiomyocytes	Result in IR^37^
Gonadal WAT of HFD mice	Exosomes	miR‐222	↑	IRS1	Liver, skeletal muscle	Impair insulin sensitivity and glucose intolerance^111^
Liver						
Hepatocytes of adult obese mice	Exosomes	miR‐130a‐3p	↓	PHLPP2	Adipocytes	Impair glucose tolerance^41^
Muscle						
Muscle cells of mice after HIIT	Exosomes	*miR‐133a/b	↑	FOXO1	Hepatocytes	Improve insulin sensitivity and decrease levels of triglycerides^112^
Lipid‐induced IR muscle cells	Exosome‐like vesicles	miR‐16	↑	PTCH1	MIN6B1 cells	Participate in adaptations of β cell mass during IR^42^
Bone marrow						
Bone marrow cells	Exosomes	*miR‐106b‐5p, miR‐222‐3p	↑	Cip/Kip family	β cells	Facilitate β cell regeneration^75^
M2‐like BMDMs	Exosomes	*miR‐690	↑	NADK	Liver, muscle, adipose tissue	Improve insulin sensitivity^76^
BM‐MSCs of aged mouse	Exosomes	miR‐29‐3p	↑	SIRT1	Adipocytes, cardiomyocytes, hepatocytes	Result in IR^43^

Abbreviations: ATM, adipose tissue macrophage; BAT, brown adipose tissue; BMDM, bone marrow‐derived macrophage; BM‐MSCs, bone marrow mesenchymal stem cells; FOXO1, forkhead box O1; FGF21, fibroblast growth factor 21; HFD, high‐fat diet; HSP60, heat shock protein 60; HIIT, high‐intensity interval training; IR, insulin resistance; IRS1, Insulin receptor substrate 1; NDUFA4, NADH dehydrogenase ubiquinone 1 alpha subcomplex 4; NADK, NAD kinase; PPARδ, peroxisome proliferator‐activated receptor δ; PHLPP2, PH domain and leucine‐rich repeat protein phosphatase 2; PTCH1, Patched 1; PPARγ, peroxisome proliferator‐activated receptor γ; SIRT2, Sirtuin 2; SOCS1, suppressor of cytokine signaling 1; SIRT1, Sirtuin 1; TRAF3, TNF receptor‐associated factor 3; WAT, white adipose tissue.

* miRNAs exerting beneficial effects.

#### 3.2.1 EV‐miRNAs from the pancreas

EV‐miRNAs derived from diverse pancreatic cells dictate T2D pathophysiology in an endocrine or paracrine manner, affecting insulin resistance and deficiency respectively. To illustrate their IR‐related effects, Xu et al reported that β cell‐derived exosomal miR‐26a could ameliorate obesity‐associated IR in hepatocytes. However, with miR‐26a expression downregulated in serum exosomes of obese patients and mice,^33^ there exists a concomitant decrease in insulin sensitivity.

Moreover, in terms of insulin insufficiency, significant effects of various pancreatic cell‐derived miRNAs were proved. Guay et al established a well‐designed cell experiment, in which untreated MIN6B1 cells were incubated with exosomes isolated from cytokine‐treated MIN6B1 cell cultures. The untreated recipient cells were induced apoptosis, with miR‐7, miR‐29a, and miR‐146a identified as factors exerting apoptosis‐evoking effects in these exosomes.^34^ In vivo studies are required to further elaborate on this issue. Also, exosomes released by inflammatory pancreatic M1‐like macrophages of high‐fat diet (HFD)‐fed mice are enriched in miR‐212‐5p. This miRNA suppresses Sirtuin 2 (SIRT2) expression and regulates the Akt pathway in recipient β cells, resulting in impaired insulin secretion.^35^

#### 3.2.2 EV‐miRNAs from adipose tissue

Aside from producing adipokines, adipose tissue interacts with other organs and influences diabetes progression via secreting EV‐miRNAs. As the primary source of EV‐miRNAs,^36^ adipose tissue plays a considerable role, mainly correlating with insulin sensitivity. For instance, hypertrophic adipocytes secret exosomes containing overexpressed miR‐802‐5p, which targets heat shock protein 60 (HSP60) directly and impairs insulin sensitivity of neonatal rat ventricular myocytes.^37^ In addition to its link with oxidative stress and inflammatory states, HSP60 deficiency causes insulin resistance and lessens the insulin‐sensitizing properties of adiponectin. Adipose tissue macrophages (ATMs) are also miRNA‐producing cells mediating IR, as observed in a classic study by Ying et al. In their experiment, miR‐155‐enriched exosomes from obese mice ATMs were administered to lean mice, resulting in poor glucose tolerance and IR.^38^ This is mainly accomplished by targeting peroxisome proliferator‐activated receptor‐γ and suppressor of cytokine signaling 1 (SOCS1).^38^, ^39^ Importantly, the knockdown of Drosha (an enzyme essential for miRNA synthesis) protected against exosome‐mediated reduction in adipocyte glucose uptake, lending credence to the notion that miRNAs represent a key functional component of these exosomes.^38^

Brown adipose tissue (BAT) represents a significant organ with metabolic benefits due to its thermogenic capability.^40^ Other than increasing energy expenditure, BAT has also been reported to increase insulin sensitivity in peripheral tissues by secreting EV‐miRNAs. In a pioneer study by Thomou et al in 2017, miR‐99b produced in mouse BAT was proved to inhibit the expression of fibroblast growth factor 21 in hepatocytes via exosomes, thus improving glucose tolerance.^36^

#### 3.2.3 EV‐miRNAs from the liver

Hepatocyte‐derived miR‐130a‐3p can be transported to adipocytes and attenuate glucose intolerance by repressing PH domain and leucine‐rich repeat protein phosphatase 2 expression in adipocytes and aiding glucose transporter 4 translocation to the membrane.^41^

#### 3.2.4 EV‐miRNAs from the muscle

Muscle cells produce EVs with distinct miRNA cargos under different conditions. For example, lipid‐induced IR muscle cells secrete miR‐16 wrapped in exosome‐like vesicles, targeting Patched 1 in MIN6B1 cells and participating in the adaptive increase of β cell mass during the IR process.^42^ This effect may suggest the role of IR in the change of β cell function, establishing a link between the two major pathophysiological changes in T2D. In contrast, exosomes from muscle cells of mice after exercise possess overexpressed miR‐133a/b, which can improve insulin sensitivity and reduce triglyceride deposits in hepatocytes.

#### 3.2.5 EV‐miRNAs from bone marrow

Series of cells from bone marrow release EV‐miRNAs concerning diabetic pathophysiology. Nanoscale exosomes generated by aged mice's bone marrow mesenchymal stem cells (BM‐MSCs) can be taken up by adipocytes, myocytes and hepatocytes, resulting in insulin sensitivity impairment both in vivo and in vitro. MiR‐29b‐3p is significantly increased in these exosomes and is the major contributor to IR, with Sirtuin 1 (SIRT1) being its downstream target.^25^, ^43^

## 4 EV‐miRNAs AND DIABETIC COMPLICATIONS

Diabetes is a chronic disease, and its hazards also come from multi‐organ complications. Long‐term hyperglycemia causes damage to the vascular system and results in microvascular disorders (e.g., retinopathy and nephropathy) and macrovascular disorders^44^, ^45^ (e.g., cerebrovascular and cardiovascular diseases, well summarized in other reviews^46^, ^47^). T2D also causes cognitive decline since brain regions are vulnerable to glucose homeostasis.^48^ Particular miRNA changes seem to predict complication occurrence and are emerging as pivotal regulators in the development of T2D complications. Further study of the nature of extracellular miRNAs for cross‐organ communication may provide insights into the pathophysiology of T2D complications.

### 4.1 Diabetes‐related endothelial cell dysfunction

Endothelial cell (EC) dysfunction is a major determinant of vascular pathogenesis. Under diabetic conditions, ECs release altered miRNA profiles in endothelial microparticles (EMPs, a type of EVs), conveying information related to vascular damage. For instance, miR‐126 is found highly enriched in EMPs in homeostatic status and can affect other ECs either via paracrine secretion or circulation.^49^, ^50^, ^51^ MiR‐126 augments the C‐X‐C motif chemokine ligand 12 (CXCL12, which counteracts apoptosis) production and endothelial progenitor cell recruitment, all of which contribute to vascular protection.^20^, ^49^ However, miR‐126 expression is significantly down‐regulated in circulating EMPs from diabetic patients and high glucose (HG)‐treated ECs,^50^, ^51^, ^52^ dampening its protective effect and facilitating vascular impairment. Additionally, the anti‐inflammatory effect of miR‐222 on the vascular system through inhibition of intercellular adhesion molecule‐1 (ICAM‐1) is compromised in a similar way.^53^, ^54^ Restoring expressions of these protective miRNAs in EVs may widen the horizons for vascular disease treatment options.

### 4.2 Diabetic retinopathy (DR)

DR, caused by abnormal retinal vasculature, is the major driver of visual impairment and blindness for working‐age individuals.^55^, ^56^

MiRNAs might be intriguing indicators for DR prediction and stratification. To illustrate, the negative correlation between miR‐15a levels in EVs and ganglion cell complex thickness (an early hallmark of retinal deterioration) in T2D patients indicates a potential role for miR‐15a in monitoring early retinal damage.^57^ In terms of DR stratification, the significantly different serum levels of miR‐210 in PDR patients, non‐PDR patients, diabetes patients without DR and healthy individuals provide a potential label to stratify retinopathy.^58^ Also, Qing et al. reported that labeling proliferative DR (PDR) with the combined detection of miR‐21, miR‐181c and miR‐1179 was 82.6% accurate.^56^

Mounting evidence suggests that circulating EV‐miRNAs may help interpret the mechanisms underlying DR occurrence, notably in retinal cell dysfunction. For example, in a hyperglycemic environment, exosomes produced by INS‐1 cells induce miR‐15a overexpression in retinal Muller cells, which can harm the retina by targeting Akt3 and causing oxidative stress.^44^ Studies on HFD and β cell‐specific miR‐15a^−/−^ mice corroborate the applicability of these findings in vivo. Likewise, the exosomal expressions of anti‐angiogenic miR‐20a/b and miR‐106a‐5p decrease in early‐stage DR mice and HG‐induced retinal photoreceptor cells, leading to neovascularization.^59^ MiR‐20b‐5p has also been proved to reduce the expression of tight junction‐related proteins in retinal endothelial cells and increase blood−retina barrier permeability, thus causing microvascular leakage.^60^ Both aberrant angiogenesis and vascular leakage lead to DR deterioration.

### 4.3 Diabetic nephropathy (DN)

DN is another common diabetic complication affecting ~40% of people with diabetes and is the main global contributor to chronic kidney disease.^61^, ^62^

Changes in urinary exosomal miRNAs, such as overexpression of miR‐19b, miR‐133b and miR‐320c, are especially indicative of renal pathology.^63^, ^64^, ^65^ These miRNA levels change before albuminuria occurrence and correlate with parameters such as HbA1c, blood pressure, serum creatinine and estimated glomerular filtration rate (eGFR).^65^ Likewise, DN severity can be assessed by miRNAs as miR‐130b and miR‐126 levels in descending order were observed in the serum of T2D patients with non‐, micro‐ and macro‐albuminuria.^66^, ^67^ To summarize, these miRNAs present as particular biomarkers in DN, providing a platform for DN prediction and stratification.

In terms of DN pathophysiology, various animal and cellular DN models have confirmed the significant roles of exosomal miRNAs. For instance, urinary EVs from T2D patients overexpress miR‐15b‐5p, which causes mesangial cell apoptosis by targeting B cell lymphoma 2 (BCL‐2, associated with apoptotic signal pathway and renal cell death).^68^ Moreover, the tubular epithelial cell‐derived exosomal miR‐19b‐3p (notably increased in DN patients and mice) is internalized by macrophages, where it causes M1‐like activation of macrophages and initiates interstitial inflammation in the kidney by directly targeting SOCS1.^64^

### 4.4 Diabetes‐related cognitive impairment

Diabetes‐related cognitive impairment is a global concern.^69^ A meta‐analysis conducted by Palta et al. indicated that compared to non‐diabetics, T2D patients exhibited poorer visual and verbal memory, processing speed, attention, executive function and motor function.^70^ These cognitive deficits may be relevant to pathological manifestations such as aberrant hippocampal neuronal activity, reduced synaptic plasticity and hyperactivated microglia.

Evaluating the alterations of brain miRNAs throughout diabetic progress is critical due to the brain's central regulatory role.^71^ MiRNAs as indications of cognitive decline in T2D patients are rarely reported. Among T2D patients, only Salama et al. identified up‐regulated miR‐132 as a predictor of mild cognitive impairment (MCI).^72^ More research is warranted to discover changed miRNAs in this illness state and to assess their potential as biomarkers.

Whereas previous studies on diabetes‐related cognitive impairment have focused inside the brain, such as brain endothelial cell‐derived miR‐126 enhancing neurological performance,^73^ communication between the brain and other organs is an attractive new research hotspot. Our group recently presented compelling evidence that, in diabetic settings, EVs secreted by dysfunctional adipocytes can be transferred to the brain, causing a massive synaptic loss in the hippocampus and compromising cognitive function. And miR‐9‐3p was considered the main component mediating the effects above by targeting brain‐derived neurotrophic factor (BDNF).^74^ These findings established a causal relationship between adipose tissue and cognitive dysfunction. And for the first time, circulating miRNAs have been shown to mediate adipose‐brain communication in diabetic circumstances. Whether these miRNAs are implicated in other diabetes‐related brain pathology demands further exploration.

## 5 miRNA‐BASED THERAPIES

### 5.1 EV‐miRNAs can be therapeutic targets of T2D

As previously mentioned, EVs and their miRNA contents can be involved in regulating key biological pathways in the development of diabetes, such as β cell function and insulin sensitivity. Targeting miRNAs involved in these pathways might contribute to T2D treatment. Herein, we list several examples to interpret EV‐miRNA functioning on β cell survival, insulin utilization or both, respectively. For instance, miR‐106b‐5p and miR‐222‐3p, which ameliorate hyperglycemia and promote β cell proliferation in diabetic mice by targeting the Cip/Kip family, are produced by bone marrow (BM) cells following bone marrow transplantation. Nevertheless, further research is needed to clarify issues including the type(s) of exosome‐secretive BM cells and the selective delivery mechanism of exosomes.^25^, ^75^ An example of miRNA functioning as an insulin sensitizer is miR‐690, whose expression is highly up‐regulated in M2‐like BM‐derived macrophage (BMDM) exosomes. Ying et al. demonstrated that obese mice treated with these exosomes for 4 weeks established improved insulin sensitivity, at least partly through miR‐690 targeting of NAD kinase (NADK, inhibits insulin action and promotes inflammatory states).^76^ Moreover, the delivery of exosomal miR‐26a from β cells to recipient cells (like hepatocytes) remarkably enhances insulin sensitivity in distant tissues. And by reducing compensatory β cell replication and impeding actin remodeling, miR‐26a also mitigates hyperinsulinemia. The dual roles clearly indicate a prospective position for β cell‐derived miR‐26a in T2D treatment.^33^ All results above lead to the concept that EV‐miRNAs are emerging as attractive potential therapeutic targets for T2D.

### 5.2 EV‐miRNAs can be therapeutic targets of T2D complications

To develop novel methods of preventing and managing diabetic complications, EV‐miRNAs may hold promise. For instance, retinal pigment epithelial cell (RPEC)‐derived exosomes are internalized by human umbilical vein endothelial cells (hUVECs) and have a protective effect against PDR. Their cargo, miR‐202‐5p, inhibits hUVEC proliferation, migration and tubular formation via deactivating the transforming growth factor β (TGFβ) signaling pathway. Additionally, these miR‐202‐5p‐containing exosomes prevent HG‐induced endothelial‐to‐mesenchymal transition (EndoMT).^77^ Moreover, Safwat et al. linked adipose MSC‐derived exosome‐induced retinal healing to the up‐regulation of miR‐222, whose expression is reduced in diabetic rabbits.^25^ Exosomal miR‐222 targets signal transducer and activator of transcription 5A (STAT‐5A) and binds to the c‐Kit receptor, thus regulating retinal angiogenesis.^78^ Taken together, these EV‐miRNAs may offer profitable therapeutic avenues for PDR patients.

There are currently no strategies to control the progression of nephropathy in DN therapy, yet EV‐miRNAs from multiple stem cell sources may offer hope. Among them, EV‐miRNAs produced from adipose tissue‐derived stem cells (ADSCs) top the list. MiR‐215‐5p, for example, can be carried in exosomes and shuttled to podocytes, where it binds Zinc finger E‐box‐binding homeobox 2 (ZEB2). As a result, it suppresses HG‐induced epithelial‐to‐mesenchymal transition (EMT) progression and podocyte migration, hence ameliorating DN caused by podocyte injury.^53^, ^79^, ^80^ ADSCs may also protect against HG‐induced mesangial hypertrophy and renal fibrosis by delivering a variety of miRNAs such as miR‐486, miR‐26a‐5p, and miR‐125a.^81^, ^82^, ^83^ Another source of EV‐miRNAs with nephroprotective effects is bone marrow. Let‐7a and miR‐222 from BM‐MSCs, target ubiquitin‐specific protease 22 (USP22) and STAT5 respectively, consequently modulating the production of TGF‐β. Suppression of the common downstream target TGF‐β protects podocytes of DN rats and HG‐treated mesangial cells, reduces apoptosis and relieves renal fibrosis.^61^, ^84^ Aside from the aforementioned sources, urine‐derived stem cells can actually release miR‐16‐5p‐enriched exosomes that are transported into HG‐treated human podocytes and improve kidney function by decreasing the expression of vascular endothelial growth factor A (VEGFA).^32^, ^85^ Given the evidence above, EV‐miRNAs may be a viable therapy option for DN.

Studies have also confirmed the therapeutic potential of EV‐miRNAs for diabetes‐related cognitive impairment.^86^ For instance, an enriched environment encourages endogenous BMSCs to increase exosomal miR‐146a secretion, which has anti‐inflammatory effects on injured astrocytes and guards against diabetes‐related cognitive impairment.^25^, ^69^ Brain endothelial cell‐derived EVs also carry miR‐146, and can partially restore short‐term memory function when injected into the cerebral ventricles of T2D mice. Additionally, miR‐146 reduces the expression of PrP^c^, which accumulates in diabetic brain cells and contributes to cognitive dysfunction.^32^, ^87^ What's more, according to our findings, suppressing miR‐9‐3p in the hippocampus or adipose tissue dramatically alleviates diabetes‐related synaptic loss and cognitive impairment,^74^ establishing the framework for clinical translation.

## 6 CURRENT CHALLENGES AND FUTURE DIRECTIONS

As previously demonstrated, EV‐miRNAs serve as crucial regulators in T2D pathophysiology, with the potential to be biomarkers and therapeutic targets. Nonetheless, despite recent advances, techniques concerning EV‐miRNAs remain lacking, and our understanding is still in its early stages. Unraveling this yet‐to‐be‐discovered universe is a fascinating endeavor for future studies. Herein, we summarize the current challenges still facing to utilize miRNAs as therapeutic targets and propose a few possible directions for future research.

### 6.1 Current challenges of EV‐miRNAs being therapeutic targets

Since miRNAs can target various mRNAs to modulate almost every biological response of the organism, miRNA‐based therapies (miRNA mimics, anti‐miRNA oligonucleotides, miRNA‐loaded EVs, miRNA sponges, and stem cell‐derived miRNAs) are highly promising. Among the above treatments, MSC‐derived extracellular vesicles and their cargo miRNAs are emerging as an exciting research topic for the treatment of various diseases. Numerous studies have demonstrated that the therapeutic effects of MSCs are mediated in a paracrine manner through EVs.^88^ Therefore, treatment with MSC‐derived EVs is more direct and avoids the range of problems associated with MSC applications. EVs have a lower risk of immune rejection, are more stable in nature, and are easier to modify and preserve, offering good prospects for application. However, there is still a long path before its clinical trials and widespread use. Problems such as what tissue‐derived MSCs should be selected (hints may be provided later), whether MSCs of heterologous origins can be administered, how to completely avoid the risk of immune rejection, in what way EVs should be administered into the body to be more effective, what is the half‐life of EVs after administration, whether these EVs can be transported to the target organ, and how to supervise are in urgent need to be addressed. Also, the translatability of rodent data to humans needs validation.

### 6.2 Unspecified sorting mechanisms of miRNAs

It is largely unknown what determines which miRNAs are secreted and thus may serve this messenger function, or remains and concentrates in the cell of origin, where they could have their action potentiated. Recently, sorting sequences were discovered to determine EV secretion or cellular retention of miRNAs, and artificial insertion or deletion of these CELLmotifs or EXOmotifs can alter the sorting of miRNAs into EVs.^89^ Further insights into the miRNA sorting mechanisms help additional modifications of EVs and their clinical transformation.

### 6.3 Effects of pharmacological treatments on exosomal miRNAs

Various pharmacological treatments have been reported to improve disease status (such as osteosarcoma, radiation‐induced lung injury, and diabetic nephropathy^90^, ^91^, ^92^) through miRNA regulation. It was demonstrated that both telmisartan and linagliptin (dipeptidyl peptidase‐4 inhibitor, glucose‐lowering medication) induce restorative effects on anti‐fibrotic miR‐29c expression,^92^ attenuating disease progression in diabetic nephropathy. This implies that whether medication effects are partially dependent on miRNA changes and may provide directions for future research. Previous studies of our group compared effects of 9 different glucose‐lowering medications on cognitive protection (our unpublished data), and reported highly variable results. Whether these drugs are correlated with exosomal miR‐9‐3p, and influence cognitive function indirectly by affecting its secretion is a fascinating topic to investigate.

### 6.4 A view based on the targeting specificity of miRNA action

As stated above, the manners in which recipient cells can internalize EVs define how targeted and particular the uptake of EVs is. This implies that the action of its cargo miRNAs is somewhat targeting as well. Similarly, our group noticed that exosomal miRNAs from adipose tissue (AT‐exo‐miRNAs) but not the liver might target the brain.^74^ This provokes us to wonder whether the risk of morbidity and disease status in targeted tissues (like the brain) could be predicted by isolating vesicles from specific organ sources (like adipose tissue). A similar conclusion may be drawn that if only AT‐exo‐miRNAs can specifically target the brain, it would suggest that adipose tissue malfunction is indicative of brain pathology and could partially explain the pathophysiology of cognitive decline.

The potential of targeting specific organs with miRNAs broadens the horizons of our utility of miRNAs for therapeutic purposes. For example, adipose tissue is the only organ that can target the brain, offering guidance for the selection of EV sources to treat cognitive impairment. As adipose tissue is very easily approachable and is the main source of exosomal miRNAs,^36^ its genetic modification to modulate metabolic diseases by altering its composition of miRNAs and surface markers of EVs, thus affecting the ratio of miRNAs in the circulation and target organs seems to be a feasible option. Direct genetic modification of the target organ would not be necessary. Even better, the modified adipose tissue can be deleted whenever adverse effects of the introduced miRNA are observed.^31^ However, it is worth noting that the composition and proportion of miRNAs in EVs do not necessarily correspond to those in donor cells,^20^ confirming the existence of sophisticated sorting mechanisms discussed before. Therefore, additional research is required to determine how the modified miRNAs are sorted into EVs, what specific organ(s) these EVs target, how to modify EVs so that they only bind to the desired organ(s)/cell(s), whether these EVs can be uptake, and whether the multi‐targeting nature of miRNAs brings about a large number of adverse effects. Moreover, AT‐exo‐miRNAs have been found to target multiple organs (such as the brain, liver, and kidneys). Identifying miRNAs co‐altered in the pathogenesis of diabetic complications and modifying them in adipose tissue may be able to prevent or ameliorate several diabetic complications simultaneously.

## CONFLICT OF INTEREST STATEMENT

The authors declare that they have no known competing financial interests or personal relationships that could have appeared to influence the work reported in this paper.

## References

[1] IDF Atlas 10th edition . https://diabetesatlas.org/atlas/tenth-edition/. 2021.

[2] Ambros V , Bartel B , Bartel DP , et al. A uniform system for microRNA annotation. RNA. 2003;9(3):277‐279. 12592000 10.1261/rna.2183803 PMC1370393

[3] Lee RC , Feinbaum RL , Ambros V . The *C. elegans* heterochronic gene lin‐4 encodes small RNAs with antisense complementarity to lin‐14. Cell. 1993;75(5):843‐854. 8252621 10.1016/0092-8674(93)90529-y

[4] Wightman B , Ha I , Ruvkun G . Posttranscriptional regulation of the heterochronic gene lin‐14 by lin‐4 mediates temporal pattern formation in *C. elegans* . Cell. 1993;75(5):855‐862. 8252622 10.1016/0092-8674(93)90530-4

[5] Moss EG , Lee RC , Ambros V . The cold shock domain protein LIN‐28 controls developmental timing in *C. elegans* and is regulated by the lin‐4 RNA. Cell. 1997;88(5):637‐646. 9054503 10.1016/s0092-8674(00)81906-6

[6] Reinhart BJ , Slack FJ , Basson M , et al. The 21‐nucleotide let‐7 RNA regulates developmental timing in *Caenorhabditis elegans* . Nature. 2000;403(6772):901‐906. 10706289 10.1038/35002607

[7] Fire A , Xu S , Montgomery MK , Kostas SA , Driver SE , Mello CC . Potent and specific genetic interference by double‐stranded RNA in *Caenorhabditis elegans* . Nature. 1998;391(6669):806‐811. 9486653 10.1038/35888

[8] Lee RC , Ambros V . An extensive class of small RNAs in *Caenorhabditis elegans* . Science. 2001;294(5543):862‐864. 11679672 10.1126/science.1065329

[9] Lau NC , Lim LP , Weinstein EG , Bartel DP . An abundant class of tiny RNAs with probable regulatory roles in *Caenorhabditis elegans* . Science. 2001;294(5543):858‐862. 11679671 10.1126/science.1065062

[10] Lagos‐Quintana M , Rauhut R , Lendeckel W , Tuschl T . Identification of novel genes coding for small expressed RNAs. Science. 2001;294(5543):853‐858. 11679670 10.1126/science.1064921

[11] Dennis C . The brave new world of RNA. Nature. 2002;418(6894):122‐124. 12110860 10.1038/418122a

[12] Rhoades MW , Reinhart BJ , Lim LP , Burge CB , Bartel B , Bartel DP . Prediction of plant microRNA targets. Cell. 2002;110(4):513‐520. 12202040 10.1016/s0092-8674(02)00863-2

[13] Griffiths‐Jones S . The microRNA registry. Nucleic Acids Res. 2004;32:109D‐1111D. 10.1093/nar/gkh023 PMC308757 14681370

[14] Kozomara A , Birgaoanu M , Griffiths‐Jones S . miRBase: from microRNA sequences to function. Nucleic Acids Res. 2019;47(D1):D155‐D162. 30423142 10.1093/nar/gky1141 PMC6323917

[15] Krol J , Loedige I , Filipowicz W . The widespread regulation of microRNA biogenesis, function and decay. Nat Rev Genet. 2010;11(9):597‐610. 20661255 10.1038/nrg2843

[16] Saliminejad K , Khorram Khorshid HR , Soleymani Fard S , Ghaffari SH . An overview of microRNAs: biology, functions, therapeutics, and analysis methods. J Cell Physiol. 2019;234(5):5451‐5465. 30471116 10.1002/jcp.27486

[17] Brandao BB , Lino M , Kahn CR . Extracellular miRNAs as mediators of obesity‐associated disease. J Physiol. 2022;600(5):1155‐1169. 34392542 10.1113/JP280910 PMC8845532

[18] Bartel DP . MicroRNAs: genomics, biogenesis, mechanism, and function. Cell. 2004;116(2):281‐297. 14744438 10.1016/s0092-8674(04)00045-5

[19] Hammond SM . An overview of microRNAs. Adv Drug Deliv Rev. 2015;87:3‐14. 25979468 10.1016/j.addr.2015.05.001 PMC4504744

[20] Rome S . Are extracellular microRNAs involved in type 2 diabetes and related pathologies? Clin Biochem. 2013;46(10–11):937‐945. 23499584 10.1016/j.clinbiochem.2013.02.018

[21] Kim VN . MicroRNA biogenesis: coordinated cropping and dicing. Nat Rev Mol Cell Biol. 2005;6(5):376‐385. 15852042 10.1038/nrm1644

[22] Xu P , Vernooy SY , Guo M , Hay BA . The drosophila MicroRNA Mir‐14 suppresses cell death and is required for Normal fat metabolism. Curr Biol. 2003;13(9):790‐795. 12725740 10.1016/s0960-9822(03)00250-1

[23] Simeone P , Bologna G , Lanuti P , et al. Extracellular vesicles as signaling mediators and disease biomarkers across biological barriers. Int J Mol Sci. 2020;21(7):2514. 32260425 10.3390/ijms21072514 PMC7178048

[24] Stepien EL , Durak‐Kozica M , Kaminska A , et al. Circulating ectosomes: determination of angiogenic microRNAs in type 2 diabetes. Theranostics. 2018;8(14):3874‐3890. 30083267 10.7150/thno.23334 PMC6071541

[25] Chang W , Wang J . Exosomes and their noncoding RNA cargo are emerging as new modulators for diabetes mellitus. Cells. 2019;8(8):853. 31398847 10.3390/cells8080853 PMC6721737

[26] van Niel G , Carter DRF , Clayton A , Lambert DW , Raposo G , Vader P . Challenges and directions in studying cell‐cell communication by extracellular vesicles. Nat Rev Mol Cell Biol. 2022;23(5):369‐382. 35260831 10.1038/s41580-022-00460-3

[27] Zhou F , Huang L , Qu SL , et al. The emerging roles of extracellular vesicles in diabetes and diabetic complications. Clin Chim Acta. 2019;497:130‐136. 31361990 10.1016/j.cca.2019.07.032

[28] Lu CC , Ma KL , Ruan XZ , Liu BC . The emerging roles of microparticles in diabetic nephropathy. Int J Biol Sci. 2017;13(9):1118‐1125. 29104503 10.7150/ijbs.21140 PMC5666327

[29] Chen X , Ba Y , Ma L , et al. Characterization of microRNAs in serum: a novel class of biomarkers for diagnosis of cancer and other diseases. Cell Res. 2008;18(10):997‐1006. 18766170 10.1038/cr.2008.282

[30] Weber JA , Baxter DH , Zhang S , et al. The microRNA spectrum in 12 body fluids. Clin Chem. 2010;56(11):1733‐1741. 20847327 10.1373/clinchem.2010.147405 PMC4846276

[31] Mori MA , Ludwig RG , Garcia‐Martin R , Brandao BB , Kahn CR . Extracellular miRNAs: from biomarkers to mediators of physiology and disease. Cell Metab. 2019;30(4):656‐673. 31447320 10.1016/j.cmet.2019.07.011 PMC6774861

[32] Hu W , Song X , Yu H , Sun J , Zhao Y . Therapeutic potentials of extracellular vesicles for the treatment of diabetes and diabetic complications. Int J Mol Sci. 2020;21(14):5183. 32708290 10.3390/ijms21145163 PMC7404127

[33] Xu H , Du X , Xu J , et al. Pancreatic beta cell microRNA‐26a alleviates type 2 diabetes by improving peripheral insulin sensitivity and preserving beta cell function. PLoS Biol. 2020;18(2):e3000603. 32092075 10.1371/journal.pbio.3000603 PMC7058362

[34] Guay C , Menoud V , Rome S , Regazzi R . Horizontal transfer of exosomal microRNAs transduce apoptotic signals between pancreatic beta‐cells. Cell Commun Signal. 2015;13:17. 25880779 10.1186/s12964-015-0097-7 PMC4371845

[35] Qian B , Yang Y , Tang N , et al. M1 macrophage‐derived exosomes impair beta cell insulin secretion via miR‐212‐5p by targeting SIRT2 and inhibiting Akt/GSK‐3beta/beta‐catenin pathway in mice. Diabetologia. 2021;64(9):2037‐2051. 34117507 10.1007/s00125-021-05489-1

[36] Thomou T , Mori MA , Dreyfuss JM , et al. Adipose‐derived circulating miRNAs regulate gene expression in other tissues. Nature. 2017;542(7642):450‐455. 28199304 10.1038/nature21365 PMC5330251

[37] Wen Z , Li J , Fu Y , Zheng Y , Ma M , Wang C . Hypertrophic adipocyte‐derived Exosomal miR‐802‐5p contributes to insulin resistance in cardiac myocytes through targeting HSP60. Obesity (Silver Spring). 2020;28(10):1932‐1940. 32844579 10.1002/oby.22932

[38] Ying W , Riopel M , Bandyopadhyay G , et al. Adipose tissue macrophage‐derived exosomal miRNAs can modulate in vivo and in vitro insulin sensitivity. Cell. 2017;171(2):372‐384. 28942920 10.1016/j.cell.2017.08.035

[39] Zhang Y , Mei H , Chang X , Chen F , Zhu Y , Han X . Adipocyte‐derived microvesicles from obese mice induce M1 macrophage phenotype through secreted miR‐155. J Mol Cell Biol. 2016;8(6):505‐517. 27671445 10.1093/jmcb/mjw040

[40] Sakers A , De Siqueira MK , Seale P , Villanueva CJ . Adipose‐tissue plasticity in health and disease. Cell. 2022;185(3):419‐446. 35120662 10.1016/j.cell.2021.12.016 PMC11152570

[41] Wu J , Dong T , Chen T , et al. Hepatic exosome‐derived miR‐130a‐3p attenuates glucose intolerance via suppressing PHLPP2 gene in adipocyte. Metabolism. 2020;103:154006. 31715176 10.1016/j.metabol.2019.154006

[42] Jalabert A , Vial G , Guay C , et al. Exosome‐like vesicles released from lipid‐induced insulin‐resistant muscles modulate gene expression and proliferation of beta recipient cells in mice. Diabetologia. 2016;59(5):1049‐1058. 26852333 10.1007/s00125-016-3882-y

[43] Su T , Xiao Y , Xiao Y , et al. Bone marrow mesenchymal stem cells‐derived exosomal MiR‐29b‐3p regulates aging‐associated insulin resistance. ACS Nano. 2019;13(2):2450‐2462. 30715852 10.1021/acsnano.8b09375

[44] Kamalden TA , Macgregor‐Das AM , Kannan SM , et al. Exosomal MicroRNA‐15a transfer from the pancreas augments diabetic complications by inducing oxidative stress. Antioxid Redox Signal. 2017;27(13):913‐930. 28173719 10.1089/ars.2016.6844 PMC5649125

[45] Kantharidis P , Wang B , Carew RM , Lan HY . Diabetes complications: the microRNA perspective. Diabetes. 2011;60(7):1832‐1837. 21709278 10.2337/db11-0082 PMC3121430

[46] Zhao S , Wang H , Xu H , et al. Targeting the microRNAs in exosome: a potential therapeutic strategy for alleviation of diabetes‐related cardiovascular complication. Pharmacol Res. 2021;173:105868. 34481974 10.1016/j.phrs.2021.105868

[47] Prattichizzo F , Matacchione G , Giuliani A , et al. Extracellular vesicle‐shuttled miRNAs: a critical appraisal of their potential as nano‐diagnostics and nano‐therapeutics in type 2 diabetes mellitus and its cardiovascular complications. Theranostics. 2021;11(3):1031‐1045. 33391519 10.7150/thno.51605 PMC7738884

[48] Dutta BJ , Singh S , Seksaria S , Das Gupta G , Singh A . Inside the diabetic brain: insulin resistance and molecular mechanism associated with cognitive impairment and its possible therapeutic strategies. Pharmacol Res. 2022;182:106358. 35863719 10.1016/j.phrs.2022.106358

[49] Jansen F , Yang X , Hoelscher M , et al. Endothelial microparticle‐mediated transfer of MicroRNA‐126 promotes vascular endothelial cell repair via SPRED1 and is abrogated in glucose‐damaged endothelial microparticles. Circulation. 2013;128(18):2026‐2038. 24014835 10.1161/CIRCULATIONAHA.113.001720

[50] Mocharla P , Briand S , Giannotti G , et al. AngiomiR‐126 expression and secretion from circulating CD34(+) and CD14(+) PBMCs: role for proangiogenic effects and alterations in type 2 diabetics. Blood. 2013;121(1):226‐236. 23144172 10.1182/blood-2012-01-407106

[51] Zampetaki A , Kiechl S , Drozdov I , et al. Plasma microRNA profiling reveals loss of endothelial miR‐126 and other microRNAs in type 2 diabetes. Circ Res. 2010;107(6):810‐817. 20651284 10.1161/CIRCRESAHA.110.226357

[52] Jansen F , Wang H , Przybilla D , et al. Vascular endothelial microparticles‐incorporated microRNAs are altered in patients with diabetes mellitus. Cardiovasc Diabetol. 2016;15:49. 27005938 10.1186/s12933-016-0367-8 PMC4804519

[53] He X , Kuang G , Wu Y , Ou C . Emerging roles of exosomal miRNAs in diabetes mellitus. Clin Transl Med. 2021;11(6):e468. 34185424 10.1002/ctm2.468 PMC8236118

[54] Jansen F , Yang X , Baumann K , et al. Endothelial microparticles reduce ICAM‐1 expression in a microRNA‐222‐dependent mechanism. J Cell Mol Med. 2015;19(9):2202‐2214. 26081516 10.1111/jcmm.12607 PMC4568925

[55] Mastropasqua R , Toto L , Cipollone F , Santovito D , Carpineto P , Mastropasqua L . Role of microRNAs in the modulation of diabetic retinopathy. Prog Retin Eye Res. 2014;43:92‐107. 25128741 10.1016/j.preteyeres.2014.07.003

[56] Qing S , Yuan ST , Yun C , et al. Serum MiRNA biomarkers serve as a fingerprint for proliferative diabetic retinopathy. Cell Physiol Biochem. 2014;34(5):1733‐1740. 25427542 10.1159/000366374

[57] Sangalli E , Tagliabue E , Sala L , et al. Circulating MicroRNA‐15a associates with retinal damage in patients with early stage type 2 diabetes. Front Endocrinol. 2020;11:254. 10.3389/fendo.2020.00254 PMC7192007 32390950

[58] Yin C , Lin X , Sun Y , Ji X . Dysregulation of miR‐210 is involved in the development of diabetic retinopathy and serves a regulatory role in retinal vascular endothelial cell proliferation. Eur J Med Res. 2020;25(1):20. 32498701 10.1186/s40001-020-00416-3 PMC7271497

[59] Maisto R , Trotta MC , Petrillo F , et al. Resolvin D1 modulates the intracellular VEGF‐related miRNAs of retinal photoreceptors challenged with high glucose. Front Pharmacol. 2020;11:235. 32210819 10.3389/fphar.2020.00235 PMC7069219

[60] Zhu K , Hu X , Chen H , et al. Downregulation of circRNA DMNT3B contributes to diabetic retinal vascular dysfunction through targeting miR‐20b‐5p and BAMBI. EBioMedicine. 2019;49:341‐353. 31636010 10.1016/j.ebiom.2019.10.004 PMC6945224

[61] Gallo S , Gili M , Lombardo G , et al. Stem cell‐derived, microRNA‐carrying extracellular vesicles: a novel approach to interfering with mesangial cell collagen production in a hyperglycaemic setting. PloS One. 2016;11(9):e0162417. 27611075 10.1371/journal.pone.0162417 PMC5017750

[62] Barrera‐Chimal J , Lima‐Posada I , Bakris GL , Jaisser F . Mineralocorticoid receptor antagonists in diabetic kidney disease ‐ mechanistic and therapeutic effects. Nat Rev Nephrol. 2022;18(1):56‐70. 34675379 10.1038/s41581-021-00490-8

[63] Florijn BW , Duijs J , Levels JH , et al. Diabetic nephropathy alters the distribution of circulating angiogenic MicroRNAs among extracellular vesicles, HDL, and ago‐2. Diabetes. 2019;68(12):2287‐2300. 31506344 10.2337/db18-1360

[64] Lv LL , Feng Y , Wu M , et al. Exosomal miRNA‐19b‐3p of tubular epithelial cells promotes M1 macrophage activation in kidney injury. Cell Death Differ. 2020;27(1):210‐226. 31097789 10.1038/s41418-019-0349-y PMC7206053

[65] Eissa S , Matboli M , Bekhet MM . Clinical verification of a novel urinary microRNA panal: 133b, −342 and −30 as biomarkers for diabetic nephropathy identified by bioinformatics analysis. Biomed Pharmacother. 2016;83:92‐99. 27470555 10.1016/j.biopha.2016.06.018

[66] Lv C , Zhou YH , Wu C , Shao Y , Lu CL , Wang QY . The changes in miR‐130b levels in human serum and the correlation with the severity of diabetic nephropathy. Diabetes Metab Res Rev. 2015;31(7):717‐724. 25952368 10.1002/dmrr.2659

[67] Al‐Kafaji G , Al‐Mahroos G , Al‐Muhtaresh HA , Skrypnyk C , Sabry MA , Ramadan AR . Decreased expression of circulating microRNA‐126 in patients with type 2 diabetic nephropathy: a potential blood‐based biomarker. Exp Ther Med. 2016;12(2):815‐822. 27446281 10.3892/etm.2016.3395 PMC4950785

[68] Tsai YC , Kuo MC , Hung WW , et al. High glucose induces mesangial cell apoptosis through miR‐15b‐5p and promotes diabetic nephropathy by extracellular vesicle delivery. Mol Ther. 2020;28(3):963‐974. 31991106 10.1016/j.ymthe.2020.01.014 PMC7054723

[69] Kubota K , Nakano M , Kobayashi E , et al. An enriched environment prevents diabetes‐induced cognitive impairment in rats by enhancing exosomal miR‐146a secretion from endogenous bone marrow‐derived mesenchymal stem cells. PloS One. 2018;13(9):e0204252. 30240403 10.1371/journal.pone.0204252 PMC6150479

[70] Palta P , Schneider AL , Biessels GJ , Touradji P , Hill‐Briggs F . Magnitude of cognitive dysfunction in adults with type 2 diabetes: a meta‐analysis of six cognitive domains and the most frequently reported neuropsychological tests within domains. J Int Neuropsychol Soc. 2014;20(3):278‐291. 24555960 10.1017/S1355617713001483 PMC4132660

[71] Fernandez‐Valverde SL , Taft RJ , Mattick JS . MicroRNAs in beta‐cell biology, insulin resistance, diabetes and its complications. Diabetes. 2011;60(7):1825‐1831. 21709277 10.2337/db11-0171 PMC3121441

[72] Salama II , Sami SM , Abdellatif GA , et al. Plasma microRNAs biomarkers in mild cognitive impairment among patients with type 2 diabetes mellitus. PloS One. 2020;15(7):e0236453. 32726329 10.1371/journal.pone.0236453 PMC7390351

[73] Venkat P , Cui C , Chopp M , et al. MiR‐126 mediates brain endothelial cell exosome treatment‐induced neurorestorative effects after stroke in type 2 diabetes mellitus mice. Stroke. 2019;50(10):2865‐2874. 31394992 10.1161/STROKEAHA.119.025371 PMC6756941

[74] Wang J , Li L , Zhang Z , et al. Extracellular vesicles mediate the communication of adipose tissue with brain and promote cognitive impairment associated with insulin resistance. Cell Metab. 2022;34(9):1264‐1279. 36070680 10.1016/j.cmet.2022.08.004

[75] Tsukita S , Yamada T , Takahashi K , et al. MicroRNAs 106b and 222 improve hyperglycemia in a mouse model of insulin‐deficient diabetes via pancreatic beta‐cell proliferation. EBioMedicine. 2017;15:163‐172. 27974246 10.1016/j.ebiom.2016.12.002 PMC5233820

[76] Ying W , Gao H , Dos Reis FCG , et al. MiR‐690, an exosomal‐derived miRNA from M2‐polarized macrophages, improves insulin sensitivity in obese mice. Cell Metab. 2021;33(4):781‐790. 33450179 10.1016/j.cmet.2020.12.019 PMC8035248

[77] Gu S , Liu Y , Zou J , et al. Retinal pigment epithelial cells secrete miR‐202‐5p‐containing exosomes to protect against proliferative diabetic retinopathy. Exp Eye Res. 2020;201:108271. 33007305 10.1016/j.exer.2020.108271

[78] Safwat A , Sabry D , Ragiae A , Amer E , Mahmoud RH , Shamardan RM . Adipose mesenchymal stem cells‐derived exosomes attenuate retina degeneration of streptozotocin‐induced diabetes in rabbits. J Circ Biomark. 2018;7:1849454418807827. 30397416 10.1177/1849454418807827 PMC6207964

[79] Jin J , Wang Y , Zhao L , Zou W , Tan M , He Q . Exosomal miRNA‐215‐5p derived from adipose‐derived stem cells attenuates epithelial‐mesenchymal transition of podocytes by inhibiting ZEB2. Biomed Res Int. 2020;2020:2685305‐2685314. 32149094 10.1155/2020/2685305 PMC7057016

[80] Peng L , Chen Y , Shi S , Wen H . Stem cell‐derived and circulating exosomal microRNAs as new potential tools for diabetic nephropathy management. Stem Cell Res Ther. 2022;13(1):25. 35073973 10.1186/s13287-021-02696-w PMC8785577

[81] Jin J , Shi Y , Gong J , et al. Exosome secreted from adipose‐derived stem cells attenuates diabetic nephropathy by promoting autophagy flux and inhibiting apoptosis in podocyte. Stem Cell Res Ther. 2019;10(1):95. 30876481 10.1186/s13287-019-1177-1 PMC6419838

[82] Duan Y , Luo Q , Wang Y , et al. Adipose mesenchymal stem cell‐derived extracellular vesicles containing microRNA‐26a‐5p target TLR4 and protect against diabetic nephropathy. J Biol Chem. 2020;295(37):12868‐12884. 32580945 10.1074/jbc.RA120.012522 PMC7489897

[83] Hao Y , Miao J , Liu W , Cai K , Huang X , Peng L . Mesenchymal stem cell‐derived exosomes carry MicroRNA‐125a to protect against diabetic nephropathy by targeting histone deacetylase 1 and downregulating Endothelin‐1. Diabetes Metab Syndr Obes. 2021;14:1405‐1418. 33790607 10.2147/DMSO.S286191 PMC8006976

[84] Mao R , Shen J , Hu X . BMSCs‐derived exosomal microRNA‐let‐7a plays a protective role in diabetic nephropathy via inhibition of USP22 expression. Life Sci. 2021;268:118937. 33347877 10.1016/j.lfs.2020.118937

[85] Duan YR , Chen BP , Chen F , et al. Exosomal microRNA‐16‐5p from human urine‐derived stem cells ameliorates diabetic nephropathy through protection of podocyte. J Cell Mol Med. 2021;25(23):10798‐10813. 31568645 10.1111/jcmm.14558 PMC8642687

[86] Zhao W , Zhang H , Yan J , Ma X . An experimental study on the treatment of diabetes‐induced cognitive disorder mice model with exosomes deriving from mesenchymal stem cells (MSCs). Pak J Pharm Sci. 2019;32(5):1965‐1970. 31813859

[87] Kalani A , Chaturvedi P , Maldonado C , et al. Dementia‐like pathology in type‐2 diabetes: a novel microRNA mechanism. Mol Cell Neurosci. 2017;80:58‐65. 28219659 10.1016/j.mcn.2017.02.005 PMC5432966

[88] Li FX , Lin X , Xu F , et al. The role of mesenchymal stromal cells‐derived small extracellular vesicles in diabetes and its chronic complications. Front Endocrinol. 2021;12:780974. 10.3389/fendo.2021.780974 PMC8721875 34987478

[89] Garcia‐Martin R , Wang G , Brandão BB , et al. MicroRNA sequence codes for small extracellular vesicle release and cellular retention. Nature. 2022;601(7893):446‐451. 34937935 10.1038/s41586-021-04234-3 PMC9035265

[90] Yang D , Chen Y , He ZNT , et al. Indoleamine 2,3‐dioxygenase 1 promotes osteosarcoma progression by regulating tumor‐derived exosomal miRNA hsa‐miR‐23a‐3p. Front Pharmacol. 2023;14:1194094. 37284323 10.3389/fphar.2023.1194094 PMC10239870

[91] Zhang N , Song GY , Hu YJ , et al. Analysis of lncRNA‐miRNA‐mRNA expression in the troxerutin‐mediated prevention of radiation‐induced lung injury in mice. J Inflamm Res. 2023;16:2387‐2399. 37292381 10.2147/JIR.S397327 PMC10246569

[92] Delic D , Wiech F , Urquhart R , et al. Linagliptin and telmisartan induced effects on renal and urinary exosomal miRNA expression in rats with 5/6 nephrectomy. Sci Rep. 2020;10(1):3373. 32099009 10.1038/s41598-020-60336-4 PMC7042229

[93] Kong L , Zhu J , Han W , et al. Significance of serum microRNAs in pre‐diabetes and newly diagnosed type 2 diabetes: a clinical study. Acta Diabetol. 2011;48(1):61‐69. 20857148 10.1007/s00592-010-0226-0

[94] Karolina DS , Tavintharan S , Armugam A , et al. Circulating miRNA profiles in patients with metabolic syndrome. J Clin Endocrinol Metab. 2012;97(12):E2271‐E2276. 23032062 10.1210/jc.2012-1996

[95] Liu Y , Gao G , Yang C , et al. The role of circulating microRNA‐126 (miR‐126): a novel biomarker for screening prediabetes and newly diagnosed type 2 diabetes mellitus. Int J Mol Sci. 2014;15(6):10567‐10577. 24927146 10.3390/ijms150610567 PMC4100169

[96] Bacon S , Engelbrecht B , Schmid J , et al. MicroRNA‐224 is readily detectable in urine of individuals with diabetes mellitus and is a potential indicator of Beta‐cell demise. Genes (Basel). 2015;6(2):399‐416. 26110317 10.3390/genes6020399 PMC4488671

[97] Higuchi C , Nakatsuka A , Eguchi J , et al. Identification of circulating miR‐101, miR‐375 and miR‐802 as biomarkers for type 2 diabetes. Metabolism. 2015;64(4):489‐497. 25726255 10.1016/j.metabol.2014.12.003

[98] Parrizas M , Brugnara L , Esteban Y , et al. Circulating miR‐192 and miR‐193b are markers of prediabetes and are modulated by an exercise intervention. J Clin Endocrinol Metab. 2015;100(3):E407‐E415. 25532038 10.1210/jc.2014-2574

[99] Seyhan AA , Nunez Lopez YO , Xie H , et al. Pancreas‐enriched miRNAs are altered in the circulation of subjects with diabetes: a pilot cross‐sectional study. Sci Rep. 2016;6:31479. 27558530 10.1038/srep31479 PMC4997329

[100] Yan S , Wang T , Huang S , et al. Differential expression of microRNAs in plasma of patients with prediabetes and newly diagnosed type 2 diabetes. Acta Diabetol. 2016;53(5):693‐702. 27039347 10.1007/s00592-016-0837-1

[101] Jones A , Danielson KM , Benton MC , et al. miRNA signatures of insulin resistance in obesity. Obesity (Silver Spring). 2017;25(10):1734‐1744. 28834285 10.1002/oby.21950 PMC5614819

[102] Yu Y , Du H , Wei S , et al. Adipocyte‐derived Exosomal MiR‐27a induces insulin resistance in skeletal muscle through repression of PPARgamma. Theranostics. 2018;8(8):2171‐2188. 29721071 10.7150/thno.22565 PMC5928879

[103] Jimenez‐Lucena R , Camargo A , Alcala‐Diaz JF , et al. A plasma circulating miRNAs profile predicts type 2 diabetes mellitus and prediabetes: from the CORDIOPREV study. Exp Mol Med. 2018;50(12):1‐12. 10.1038/s12276-018-0194-y PMC6312530 30598522

[104] La Sala L , Mrakic‐Sposta S , Tagliabue E , et al. Circulating microRNA‐21 is an early predictor of ROS‐mediated damage in subjects with high risk of developing diabetes and in drug‐naive T2D. Cardiovasc Diabetol. 2019;18(1):18. 30803440 10.1186/s12933-019-0824-2 PMC6388471

[105] Parrizas M , Mundet X , Castano C , et al. miR‐10b and miR‐223‐3p in serum microvesicles signal progression from prediabetes to type 2 diabetes. J Endocrinol Invest. 2020;43(4):451‐459. 31721085 10.1007/s40618-019-01129-z

[106] Šimonienė D , Stukas D , Daukša A , Veličkienė D . Clinical role of serum miR107 in type 2 diabetes and related risk factors. Biomolecules. 2022;12(4):558. 35454146 10.3390/biom12040558 PMC9027608

[107] Sun Y , Zhou Y , Shi Y , et al. Expression of miRNA‐29 in pancreatic beta cells promotes inflammation and diabetes via TRAF3. Cell Rep. 2021;34(1):108576. 33406428 10.1016/j.celrep.2020.108576

[108] Wang L , Zhang B , Zheng W , et al. Exosomes derived from pancreatic cancer cells induce insulin resistance in C2C12 myotube cells through the PI3K/Akt/FoxO1 pathway. Sci Rep. 2017;7(1):5384. 28710412 10.1038/s41598-017-05541-4 PMC5511275

[109] Liu T , Sun YC , Cheng P , Shao HG . Adipose tissue macrophage‐derived exosomal miR‐29a regulates obesity‐associated insulin resistance. Biochem Biophys Res Commun. 2019;515(2):352‐358. 31153636 10.1016/j.bbrc.2019.05.113

[110] Tian F , Tang P , Sun Z , et al. miR‐210 in exosomes derived from macrophages under high glucose promotes mouse diabetic obesity pathogenesis by suppressing NDUFA4 expression. J Diabetes Res. 2020;2020:6894684‐6894612. 32258168 10.1155/2020/6894684 PMC7106924

[111] Li D , Song H , Shuo L , et al. Gonadal white adipose tissue‐derived exosomal MiR‐222 promotes obesity‐associated insulin resistance. Aging. 2020;12(22):22719‐22743. 33197889 10.18632/aging.103891 PMC7746358

[112] Castano C , Mirasierra M , Vallejo M , Novials A , Parrizas M . Delivery of muscle‐derived exosomal miRNAs induced by HIIT improves insulin sensitivity through down‐regulation of hepatic FoxO1 in mice. Proc Natl Acad Sci U S A. 2020;117(48):30335‐30343. 33199621 10.1073/pnas.2016112117 PMC7720135

